# Palm Oil Fuel Ash-Based Eco-Efficient Concrete: A Critical Review of the Short-Term Properties

**DOI:** 10.3390/ma14020332

**Published:** 2021-01-11

**Authors:** Mugahed Amran, Gunasekaran Murali, Roman Fediuk, Nikolai Vatin, Yuriy Vasilev, Hakim Abdelgader

**Affiliations:** 1 Department of Civil Engineering, College of Engineering, Prince Sattam Bin Abdulaziz University, Alkharj 11942, Saudi Arabia; 2 Department of Civil Engineering, Faculty of Engineering and IT, Amran University, Amran 9677, Yemen; 3 School of Civil Engineering, SASTRA Deemed University, Thanjavur 613401, India; murali_220984@yahoo.co.in; 4 Polytechnic Institute, Far Eastern Federal University, Sukhanova Str., 690922 Vladivostok, Russia; roman44@yandex.ru; 5 Higher School of Industrial, Civil and Road Construction, Peter the Great St. Petersburg Polytechnic University, 195251 St. Petersburg, Russia; vatin@mail.ru; 6 Department of Road-Building Materials, Moscow Automobile and Road Construction University, 125319 Moscow, Russia; yu.vasilev@madi.ru; 7 Department of Civil engineering, Faculty of Engineering, University of Tripoli, Tripoli 83038, Libya; h.abdelgader@uot.edu.ly; 8 Faculty of Civil and Environmental Engineering, Gdańsk University of Technology, 80-233 Gdańsk, Poland

**Keywords:** palm oil fuel ash, applications, chemical composition, clean production, short-term properties, eco-efficient concrete

## Abstract

The huge demand for concrete is predicted to upsurge due to rapid construction developments. Environmental worries regarding the large amounts of carbon dioxide emanations from cement production have resulted in new ideas to develop supplemental cementing materials, aiming to decrease the cement volume required for making concrete. Palm-oil-fuel-ash (POFA) is an industrial byproduct derived from palm oil waste’s incineration in power plants’ electricity generation. POFA has high pozzolanic characteristics. It is highly reactive and exhibits satisfactory micro-filling ability and unique properties. POFA is commonly used as a partially-alternated binder to Portland cement materials to make POFA-based eco-efficient concrete to build building using a green material. This paper presents a review of the material source, chemical composition, clean production and short-term properties of POFA. A review of related literature provides comprehensive insights into the potential application of POFA-based eco-efficient concrete in the construction industry today.

## 1. Introduction

The current concrete is generally categorized as the second utmost extensively use building material following water and has a global consumption percentage of approximately 25 × 10^9^ tons per year [1]. Concrete is characterized by its low cost, durability, source of binder materials, and formability to any form [2,3]. Binding methods and constituents utilized for concrete production are crucial in concrete technology to set, harden and adhere all materials together [4]. For example, cement deems the greatest widely utilized paste materials in concrete structures with around 3.3 billion tons as an annual cement usage [3]. The worldwide making of ordinary Portland cement (OPC) upsurges by 9% annually and poses an essential ecological issue because it releases a considerable volume of CO_2_ into the air [5]. In particular, OPC production emits approximately 1.5 billion tons of greenhouse gases with an annual average of 6% of the total emissions of several industries worldwide [5,6]. The greenhouse influence avoids replicating solar radioactivity back into the atmosphere and thus maintains the average heat on Earth surface between 15 and 18 °C [7]. The concentration of CO_2_ in the air augmented by 30% (467 million tons (Mt)) in 2013, as compared to 2012 [7,8].

**Figure 1:**
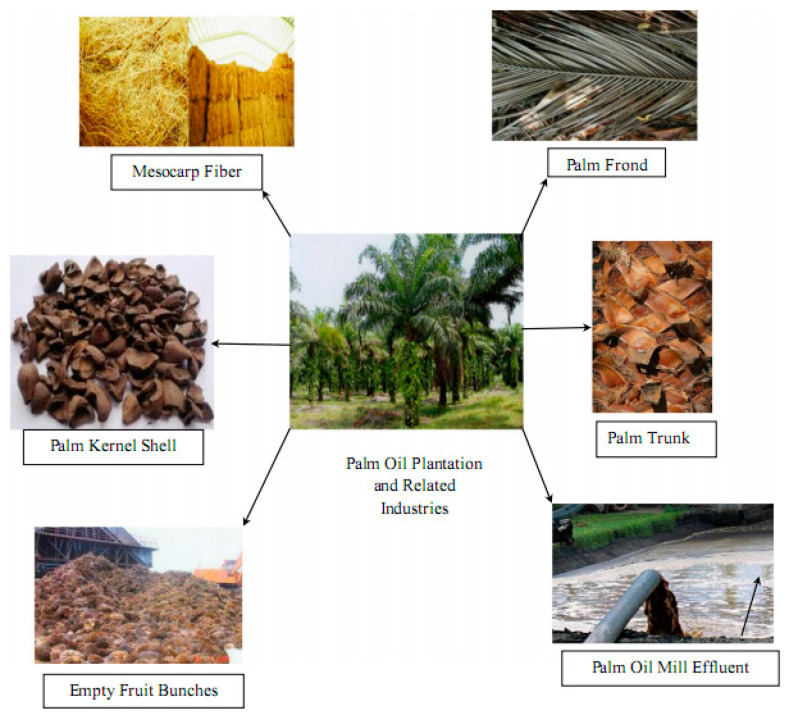
Malaysian palm oil plantation and industry [10]. Reprinted with permission from Elsevier [10].

**Figure 2:**
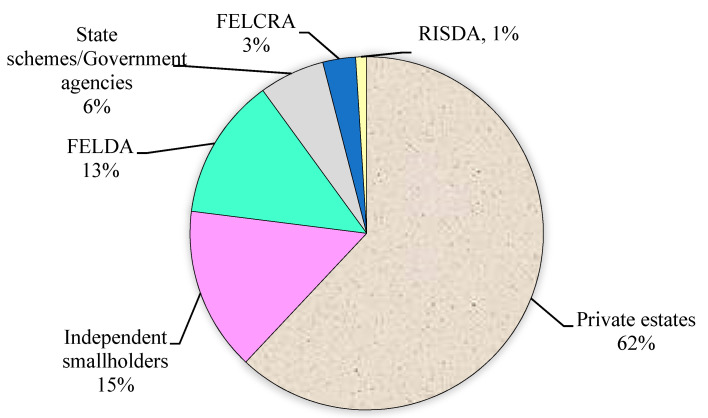
Percentage of plantation ownership of oil palm in Malaysia [12]. Reprinted with permission from Elsevier [12].

However, incorporating supplementary cementing material (SCM) decreases cement in concrete; consequently, this sustainable construction method lessens greenhouse gas emissions, saves energy, reduces energy consumption, and protects natural resources [9]. Palm oil fuel ash (POFA) is a by-product derived due to the combustion of resides from a palm oil (PO) tree (Figure 1) in the palm industry [10]. In Malaysia (Figure 2) palm oil cultivation was restricted to 54 thousand hectares in the year 1960 and considerably increased to 5390 × 10^3^ hectares in the year 2014. In Indonesia, farming of PO was confined to 6500 × 10^3^ hectares in 2012 [11]. At present, PO agriculture is the primary source of the monetary growth of both states. The primary production of PO is found in Indonesia and Malaysia with approximate rates of 86% and 14%, respectively, as reported by an international statistic in 2011 [12,13]. For example, the *Elaeis Guineensis* is a PO tree known as an ornamental plant in 1870 in the southeast of Asia countries (e.g., 3 million hectares in Malaysia) [11,14]. In PO mill plants, around 90 × 10^6^ tons of PO plantation are produced annually and burnt after removing oil from the mushy palm product at 800–1000 °C as heater fuel to create mist for electricity production and extraction of PO [15]. POFA is considered a hazardous waste to the environment. The continuous increase in PO production in tropical states has led to a cumulate quantity of POFA and creates a substantial ecological load [11]. In Malaysia, scholars have investigated the potential of POFA as fertilizer for agriculture [15]. However, POFA is discarded in open spaces beside PO mills, deprived of any profitable return due to the deficiency of adequate nutrients to be utilized as manure, thereby instigating ecological pollution and human health risk [16]. To overcome this problem, numerous investigations were directed to study the possibility of using POFA in making durable concrete. In Malaysia, properly processed POFA was first used successfully as a partial-cement-replacement (PCR) to make concrete in 1990 [16,17,18]. POFA used within 10–60% replacement by weight of cement does not affect concrete strength and exhibits durability comparable with OPC concrete. However, the most suitable POFA content added in concrete is 30% [19,20,21]. POFA concrete exhibits good strength and adequate durability as it can suppress the expansion of alkali-silica reaction [19,20] and external sulfate attack [22,23]. POFA is utilized to manufacture ordinary, aerated and ultra-high-strength concretes due to significant variances in surface fineness, particle size, and pozzolanic activity (PA) [16,24,25].

Several scholars attempted to reduce the utilization of OPC by producing environment-friendly concrete that uses certain by-product materials [14,16,23,26,27]. The construction industry in Southeast Asian countries has begun to demand increased production of POFA because of its crucial role in decreasing the volume of toxins and CO_2_ levels produced by OPC manufacturing. The use of PO-based biodiesel could decrease the present emissions level of greenhouse gas by almost 62% [28]. This paper presents a review of the material source, chemical composition, clean production and short-term properties of POFA. A review of related literature provides comprehensive insights into the potential application of POFA-based eco-efficient concrete in the construction industry today. Furthermore, the lack of knowledge concerning POFA-based eco-efficient concrete highlights future research needs.

## 2. POFA Source and the Possibility of Its Application in Construction

**Figure 3:**
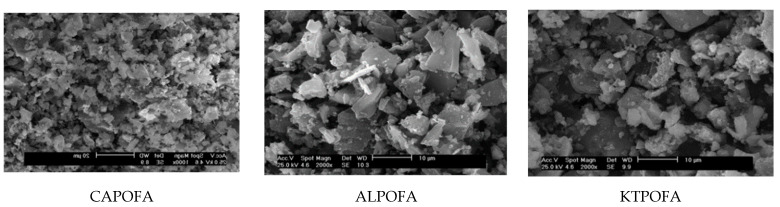
Scanning-electron-microscopy (SEM) [30]. Reprinted with permission from Elsevier [30].

POFA is an engineering byproduct [11,16,19,22,23,26] generated from the waste of the PO industry [11,12,14]. POFA is the residual of the PO fruitiness clusters after oil removal in PO mills [11,16,29]. In practice, three typical POFA materials are available in Malaysia and other Southeast Asian countries. Such materials are generated from three different palm oil mills, namely CAPOFA, KTPOFA, and ALPOFA (Figure 3) [30]. In the steam boiler, the palm kernel and husk shell are also combusted to produce POFA, containing a large amount of silica oxide content that satisfies the pozzolanic property criterion and can be potentially used as cement replacement [19,24]. In general, cultivation waste ashes encompass high silica amounts, leading to pozzolanic materials [18,31]. Nevertheless, numerous researchers stated that the use of POFA in normal concrete [29,32], as well as high-strength [11,30] and lightweight concrete, including foamed one [33,34]. However, an empty fruit bunch (EFB) is an appropriate raw material burnt in reservoirs used to produce POFA and electricity for palm oil mills. These residues are abundant and readily available within the plant areas [10,27,29]. Approximately 5% of EFB in terms of solid waste weight can be produced as a POFA [35]. Given the continuously increasing making of PO, higher volumes of POFA will be made in PO mills. Therefore, the dump of PO wastes in open areas deprived of any economic profit and irritation to the environs causes severe problems because of the failure to utilize this ash and it is disposed of in open areas without any control [10,28,30]. To resolve the ecological issues caused by POFA, numerous studies have examined the feasibility of using this POFA ash as a PCR to produce high-performance concretes [11,16,36]. Reportedly, ground POFA as a partial replacement of OPC resulted in increased water demand for concrete’s preferred workability [20]. However, the concrete strength with 20% ground-POFA is improved and decreased when the inclusion of the POFA binder exceeded 20% and increased concrete permeability [20,37,38]. Tay [39] used unground POFA as a partial substitution of OPC and reported its poor PA. Hence, POFA should not be added with contents more than 50% by weight of cement. However, the addition of 30% ground POFA content was found to slightly decrease the concrete strength at 21 days from casting time. The concrete strength with 5%–15% at and after 28 days satisfied the ASTM C 618 requirement [40]. Furthermore, Bamaga [30] used up to 40% ground POFA in concrete and found the strength, elasticity modulus, Poisson’s ratio, creep and shrinkage of concrete; findings revealed that the properties of hardened concrete with as much as 30% POFA are similar with reference specimens. Dozens of researchers stated that the potential utilization of ground POFA as an SCM in concrete production is augmented due to its significant PA [16,26,30,32].

## 3. Clean Production of POFA

**Figure 4:**
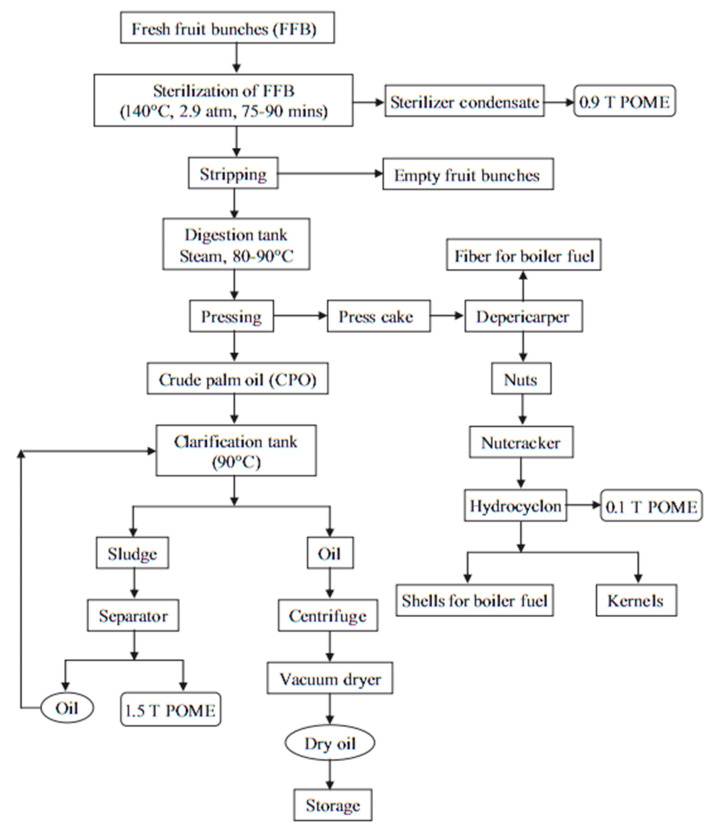
Production of typical POFA materials [42]. Reprinted with permission from Elsevier [42].

**Figure 5:**
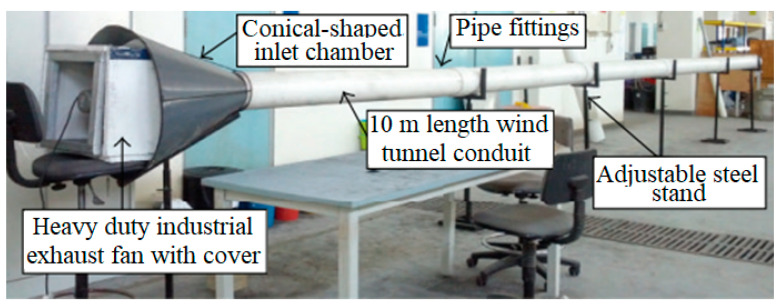
Right-side-view of the setup of wind tunnel manufacturing scheme [43]. Reprinted with permission from Ahmadi et al. [43].

In the clean production-process-of-POFA ash, large amounts of residues, such as fibers, nutshells, and EFBs, are produced from palm oil mills and burnt at heating between 800 and 1000 °C as fuels to afford steam for electricity production in the PO mills [12,16,30,39]. After the sweltering process, almost 5% of ash by-product is obtained from the residues, known as POFA and the rest are waste materials [2,41]. The 300-µm sieve is used to sieve ashes to remove undesired particles (Figure 4) [42]. The ashes are then pulverized using a milling machine to decrease the particle size to a maximum of 45 µm. The wind-tunnel-system (Figure 5) utilizes forced-air as an airstream to transmit the ground POFA in a 10-m segmental-channel, consisting of 5 linked 2-m segments. Large-sized POFAs usually settle down at the front part of the channel, while the finer POFAs are blown toward the tunnel’s end. POFA is collected and analyzed at different sections of the tunnel using a laser particle-size-analyzer (PSA), nitrogen absorption, and SEM with energy distracted X-ray (SEM-EDX). Before these tests, the utilized POFA at each segment of the wind tunnel is calcined in a muffle boiler at 500 °C to eliminate extra CO_2_ [31,36]. POFA should be sieved and ground to 45 µm or less for use as a PCR; else, core POFA is merely significant as plaster. The charge of grinding and sieving POFA could reimburse the cost of ecological risk from reducing the dust as dumping or whichever accomplishments. POFA is grayish and turns black with increasing amounts of unburned-carbon [11,16,30,32]. The POFA particle size is commonly finer than OPC [15,23,32,40]. The particle shapes of ALPOFA, KTPOFA, and CAPOFA are shown in (Figure 3) through the SEM method. The big particles of core POFA are produced in three typical shapes: irregular, thinner, and crushed, relying on the grinding process.

The refining process of POFA can be operated by selecting one kilogram of POFA and fill into a graduated cylinder [44]. The water is subsequently included in the same cylinder until 80%-full. The POFA mix and water are enthused for almost 30 min using a portable mixer. After the mixing procedure, the first water used in the POFA is removed due to filthiness. Again, water is included in the same cylinder until 80%-full and left for 24 h. After 24 h of precipitation, two main POFA layers appear, namely, layers 1 and 2 [45]. POFA layer 1 is withdrawn using a spatula because it is floating. Then, layer 2 is removed using a syringe withdrawal method. After separating the layers of POFA, they are placed in different containers. Then, POFA layers 1 and 2 are dehydrated in the kiln for approximately one day at 100 °C. Owing to the high humidity of POFA, the drying time must be 24 h and above to ensure the complete drying of POFA and avoid stickiness during the grinding process [15,44].

## 4. Chemical Composition

Mineral admixtures vary significantly in chemical compositions and cement interactions due to their variable sources and procedures [46,47,48]. Reportedly, the primary chemical compound of POFA is SiO_2_ (42–66%) and the secondary compounds are Fe_2_O_3_ and Al_2_O_3_, the loss-on-ignition (LOI, 2.4–21.5%) and SO_3_ (0.2–4 (Table 1). In most cases, LOI and Na_2_O are considerably larger than the limit stated in ASTM C 618 [21] while SO_3_ is less than that specified in the same code. In POFA, the sum of Al_2_O_3_, Fe_2_O_3_, and SiO_2_ is found almost higher or lesser than 70% due to the reduction of big particle sizes of POFA and unburned fibers. In contrast, LOI is close to 10% and SO_3_ is less than 4%, indicating not to fulfill the requirements of pozzolanic prescribed in ASTM C 618 [21,39,49]. However, in a few investigations, it is revealed that the entire volume of Fe_2_O_3_, Al_2_O_3_, and SiO_2_ of POFA is not as much as the base prerequisite for common pozzolan as indicated by ASTM C 618 [21,22]. At this cause, it is recommended to make sure that the silica oxide content in pozzolans can react with calcium hydroxide (Ca(OH)) from the hydration process. Simultaneously, the pozzolanic reactions produce more calcium silicate hydrate (C–S–H) gel compound. Moreover, these reactions also reduce the amount of calcium hydroxide. This contributes to the strength of the concrete and produces stronger and denser concrete with enhanced durability.

However, in other research findings, the amount of CaO in POFA is 4.12%, as this percentage is less than 5% as requested by Class F [28,50]. Also, POFA reportedly satisfies a pozzolanic necessity and might be characterized within Class C in line with the code determination expressed in ASTM C618 [20,21]. The chemical composition of OPC and POFA showed in Table 2, exhibiting that ALPOFA and CAPOFA have very high silica ashes and can be categorized as a class N pozzolana in line with ASTM C 618 [21]. However, LOI influence has been confirmed unproductive. KTPOFA fulfills the necessities of a class F pozzolana and the code ASTM C 618 [21]. Subsequently, the results that legitimize the classification of POFA in light of its chemical composition are disproving. Therefore, more investigations are required to address this logical inconsistency.

## 5. Physical Properties

In POFA, the physical properties are significantly affected by the boiling condition, in particular, at elevated temperatures. As POFA matures, the concrete remains to contract reliant on the density due to the constituent’s active reaction. Nevertheless, the shrinkage level rapidly reduces and persists over time. The properties are density, color, particle shape and size, fineness, PA, dry shrinkage, soundness, creep, water permeability, and porosity (Table 3).

### 5.1. Density and Specific Gravity

According to Neville [75], the common variety of densities of concrete ranges from approximately 2000 to 2300 kg/m^3^, as measured by ASTM C 567 [76], albeit, the regular density for POFA mixtures is about 2100 kg/m^3^ as it is nearly 12.5% less than that of the normal concrete [14,47,77]. In general, the concrete density also inclines to reduce with increasing substitution level of POFA [12,16,49,52,53,54,62,65,78]. The bulk density of POFA concrete may be reduced because the specific gravity (SG) of POFA is lower than the cement and the possibility of POFA to trick air bubbles. Reportedly, the replacement of POFA by weight of the binder with volume exceeding 15% reduced the concrete strength, except for the substitution volume limited to 10% or less with an average density [25,60,79]. The absolute density of unground POFA is almost 60% lesser than that of OPC due to the porosity of the POFA particle [23,43,60,69]. Ranjbar et al. [51,64] reported that the bulk densities of self-compacting concrete (SCCs) comprising 20%, 15%, and 10% POFA condensed by 5.75%, 4.96%, and 3.68%, correspondingly, in comparison with the reference samples. Moreover, the concrete consisting of 50% and 70% treated POFA exhibited more steady performance during the heat upsurge in the reference cement paste samples. This spectacle may be because of the high volume of high-density C–S–H in these specimens, having no such effect by high-temperature exposure [80]. The results are similar to the findings mentioned above reported on the effect of POFA fineness on the mixed cement adhesive microstructure [49,62,81]. Moreover, the high strength of FC within 1–43 MPa is mainly found relying on the density and use of supplementary materials [16,30,82,83,84,85].

POFA has a varying SG [11,12]. However, according to many researchers [30,32,74,86,87,88], SG does not exceed 3.0. POFA delivered from the industry is a big size of particle material having a spongy texture [11]. It is found that the SG of POFA is limited between 1.95 and 2.05. Numerous scholars [16,22,32,60,62,81] revealed that after crushing, the SG of POFA improved due to reduced porosity. The SG of POFA is lower than cement but identical to that of fly ash. However, the SG of POFA can be increased within 2.22–2.78 after the grinding process [16,74,78,89]. This result maybe because grinding decreases the porosity by lessening the particle size. Hence, OPC particles are weightier and thicker than POFA ash [19,38]. For instance, the SG of unground POFA commonly differs within 1.70–1.98 as it is a 2/5 smaller than the SG of cement [39]. The SCC containing 50% of treated POFA is slightly accomplished due to that the rate of POFA content deliberates the greater binder volume substituted in the concrete mix proportions [61].

### 5.2. Color

**Figure 6:**
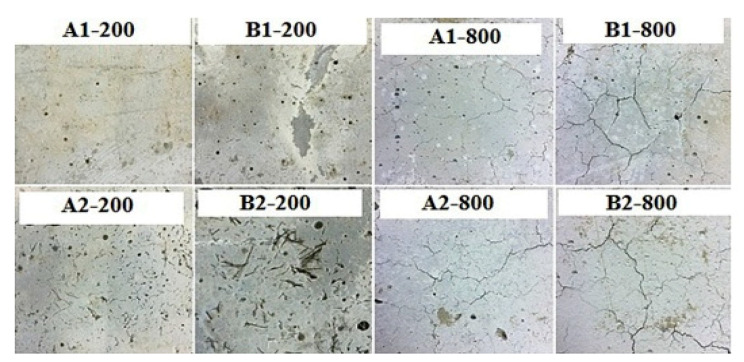
Surface texture of concrete specimens exposed to high temperatures [57]. Reprinted with permission from Elsevier [57].

POFA is generally depicted in gray and dark colors attributed to increasing amounts of unburnt carbon [11]. Even though the particles have a wide range of forms, they are majority sphere-shaped and have SG less than cement [54]. This finding may be attributed to the decrease of LOI via heating treatment [52]. The change in color of POFA concrete depends on the volume of POFA added, the chemical reaction and the degree of heating [54]. However, investigators found that the concrete color indicates the intensity and duration of heat exposure to which it was fired [16,52,53]. In particular, unground POFA has a gray color due to the unburnt carbon content left-hand at a reasonably low heat and whitish in the absence of un-burnt carbon and dark grey of ground POFA [28]. At room temperature, the surface of OPC is revealed to have a gray color and faint gray for POFA-based concrete samples with even surfaces (Figure 6). These forms were preserved at a temperature of 200 °C.

Nevertheless, at 800 °C, a light ashy color for POFA and whitish-gray color for normal concrete samples were detected [90]. Fine cracks initiated to mature at 800 °C in POFA and OPC mixtures for water-air-cooled specimens. Awal et al. [54] reported that at room temperature (27 °C), POFA and OPC concrete samples are steamy gray and black, respectively, and have even surfaces and impeccable edges. These features were conserved up to 200 °C. At 400 °C, POFA and OPC concrete samples are fawn and light black, respectively, and have even and impeccable edges during the total cooling condition [52]. At 600 °C, POFA and OPC concrete samples are ghost white and dark gray, respectively, and have rough edges [70,88]. In the normal concrete, at 800 °C, surface cracks were initiated for entirely freezing conditions and it has a whitish-gray-color. POFA-based concrete agonized from disintegration because of water freezing and propagated fine cracks from air freezing. This sample has brownish-black-gray color [16,61,78].

Another study found that POFA concrete samples exhibited grayish color, fineness, and low density at 100 °C for 24 to release moisture [73]. For clarity, the increase of POFA content blackens the samples before exposure to high temperature because POFA has a dark color [22,49,62]. However, in the wake of being presented to high temperature, the examples lightened and indicated distortion [64].

### 5.3. Particle Size and Shape

**Figure 7:**
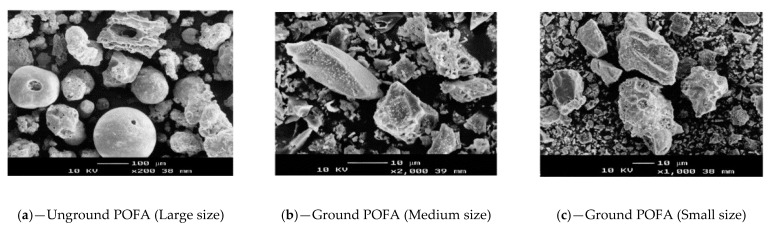
Scanning electron micrographs (SEMs) of OPC and POFA [22]. Reprinted with permission from Elsevier [22].

**Figure 8:**
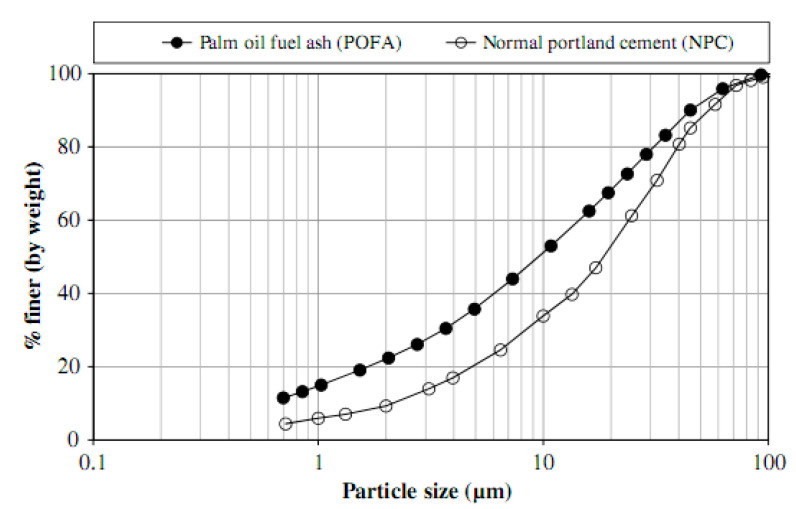
Particle size distributions of OPC and POFA [23,94]. Reprinted with permission from Elsevier [23,94].

The size and shape of particles differ between ground and unground-POFA [4,22,91]. Regarding the SEM analysis, unground POFA particles are typically spherical, large, and porous, having even surfaces, signifying complete burning (Figure 7b) [20,37,38]. Meanwhile, the ground POFA commonly contains wrinkled particles with angular and irregular shapes similar to OPC [20,22] (Figure 7a,c). Moreover, the OPC particle size is similar to that found in ground POFA (Figure 7b,c). Ground POFA has lesser particles and unground-POFA has bigger particle size than cement [22]. The distribution of the classic size of a particle of OPC and POFA is presented in Figure 8. The unground POFA median-particle-size (*d*_50_) differs between 62.5 and 183 µm. These results are more significant than that of cement (up to 20 µm) [20,38]. After grinding, the values can be decreased from 7.2 µm to 10.1 µm due to the improved POFA fineness [16,92]. From the curve of the particle–shape distribution shown in Figure 8, the following values are deduced:

D_ground POFA_ = 0.35 µm
D_OPC_ = 0.2 µm
D_unground POFA_ = 0.07 µm
C_u_ = (D_OPC_/D_ground POFA)_ = 2.86
C_c_ = (D_unground POFA_)^2^/(D_ground POFA_ × D_OPC_) = 0.35
C_u_ (uniformity coefficient) is more than 2, and C_c_ (coefficient of gradation) is limited to 0.30 and 1. Therefore, M-sand is quite rated and is within zone C BS882:1992 [93].

### 5.4. Fineness

POFA fineness is found to develop the concrete strength attributable to its density, and homogeneity [22,32,47,49,60,62,63,75,81]. Reportedly, the concrete comprising 10% and 20% treated POFA increased the concrete strength. These results probably because of the contribution of POFA that behaves as a micro-filler used to seal the voids among the particles, leading to an increase in the concrete microstructure [11,29,74]. Based on previous investigations, unground-POFA is finer than OPC and extra-fine than ground-POFA [11]. Thus, ground POFA is utilized to improve the degree of fineness and enhance the mixture’s reactivity and therefore increase concrete properties. The shape of a particle of POFA could be decreased by crushing in ball-mills and ground in a LAAM using a mild steel bar of 800 mm length and 12 mm diameter [12,19,20,32,65]. POFA is found to be less permeable with small particles after grinding [95]. In practice, the fineness of POFA is commonly measured regarding the weight of the rate passing via or being reserved in a sieve with a 45-µm opening. POFA has a larger specific surface area compared to OPC. The study reported that the rate mass of ground POFA and unground POFA that retained on sieve No. 325 is limited to 1.0–3.0% and 41.2–94.4%, respectively. Ranganath et al. [96] deliberated the effect of ash fineness on concrete strength development; coarse-particles’ utilization decreased the concrete strength. In general, POFA exhibits the utmost strength because of its high-fineness. Swamy [97] verified the effectiveness of finer 10 µm POFA in growing the concrete strength than 45 µm POFA; quite fine pozzolana is required to be naturally-extremely reactive. The efficiency of POFA with high fineness in up-surging particles reactiveness to fasten the reaction of pozzolanic and thus enhance durability to acid attack is proven, as indicated by the lower reduction ratio of POFA (10% of replacement) concrete with 10 µm POFA than that with 45 µm POFA. Moreover, the use of Ca(OH)_2_ content reduced with the increased substitution of SCM material, pozzolanic reaction and fineness [8]. This improves its sulfate resistance and the finer POFA contributed to rapid pozzolanic reaction than coarse POFA [11,22,30,38]. Another research findings revealed that the blended cement paste encompassing fly ash and silica fume ash decreased Ca(OH)_2_, albeit, the C_2_ASH_8,_ C–S–H and the mass loss of ettringite improved with protracted curing. Moreover, workability is generally decreased as the content of POFA rises due to the higher fineness of POFA, whose high surface area can absorb more water [11,30,53,94,98].

### 5.5. Heat of Hydration

POFA is employed in high volumes to decrease the heat of hydration (HoH) of concrete [31,32,47,52,58,65,75,92,99,100,101]. The sum of pozzolanic SCMs has improved with the rapid developments in concrete technology. It is reported that the concrete encompassing 100% OPC and 50%, 60%, and 70% POFA at the early-age. However, over time, concrete, including POFA revealed a reduction in the entire heat increase and overdue the highest temperature incidence (Table 4) [65,102]. This indicates that the increase in ground POFA content reduced the rise in the peak temperature of concrete [19,74].

Reportedly, the inclusion of 30% ground-POFA contributed to 15% lower temperature, showed the lowermost temperature greater than OPC concrete and decreased the total heat release [65]. This finding was due to presence of POFA as a PCR. Thus, POFA substantially decreased the entire temperature rise in concrete. Thus, the time–temperature behavior in concrete encompassing numerous volumes of ash is worth studying in the future [52,58,65].

### 5.6. Drying Shrinkage

**Figure 9:**
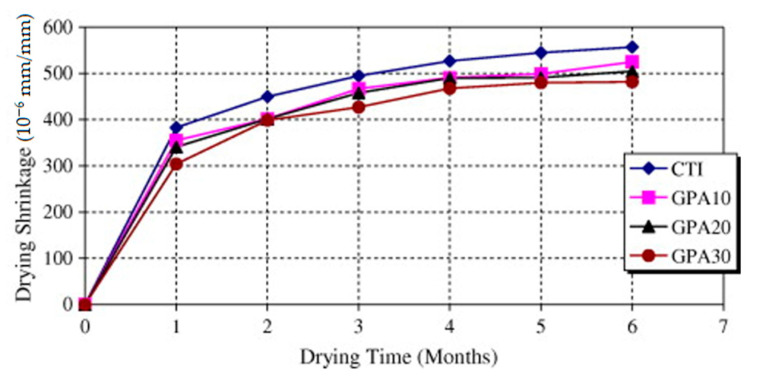
Influence of POFA on the concrete DS [104]. Reprinted with permission from Elsevier [104].

The findings of the drying shrinkage (DS) test of water- and air-cured samples are presented in Figure 9 [47,81,103]. Reportedly, the increase of unground POFA content reduced the DS slightly after 28 days [32]. The concrete DS with 10%-POFA is similar to that of control samples. The investigation reported that the mortar with 10% to 40% POFA exhibited the uppermost DS; 20% and 30% POFA gave similar DS development in control samples [32]. However, the DS of the 70%, 60% and 50% POFA mixes in 182 days are 13%, 11%, and 7%, respectively, compared with the control [61,64]. It is also revealed that the DS of the concrete encompassing 30%, 20%, and 10% POFA are 494, 505, and 525 × 10^−6^ micro-strain, correspondingly, while the control samples is 557 × 10^−6^ µm-strain at 182 days [32,49,62]. Similarly, another study reported that the DS of the concrete comprising 30%, 20%, and 10% fine POFA are 645, 670, and 707 × 10^−6^ µm-strains, correspondingly, while the control sample is 785 × 10^−6^ µm-strains [29,69,92]. However, the highest concrete strength with POFA formed lesser DS than the control sample for any volume of POFA added (Figure 9) [16,32,104]. The low value of DS in POFA concrete maybe because of the densification of concrete’s permeable structure. The inclusion of POFA lessened the pore diameters because of the refinement of pores [64]. The very fine POFA instigated pore refinement [62]. The transformation of wide pores into small pores pore via the refinement process may decrease the water loss from the concrete surface and decrease the DS [105]. Moreover, up to 30% FP of 10 μm diameter as a binder substitution condensed the concrete DS [22,60]. These findings show that POFA is an outstanding SCM used as a significant pozzolan to substitute share of OPC in concrete and mortar production with comparatively ultra-high-strength with minimal DS.

### 5.7. Porosity

**Figure 10:**
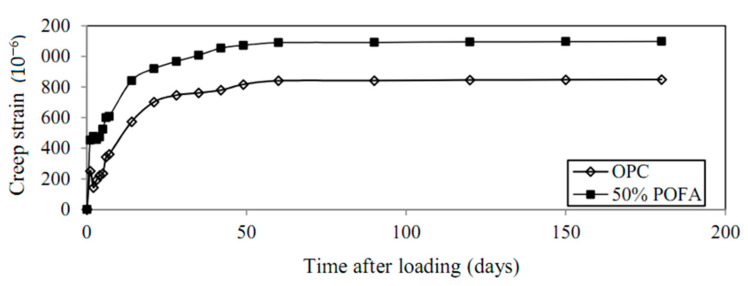
Creep strain vs. time after loading of POFA concrete incorporated with ground POFA [109]. Reprinted with permission from Trans Tech Publications [109].

Reportedly, the increase in POFA content may be caused by a high porosity due to the permeable nature of POFA [47,75,106,107]. The mercury-intrusion-porosimetry (MIP) test is utilized to investigate the porosity of concrete [92,108]. Porosity increases with the water content, which could have adverse effects on fresh material properties [51]. Several researchers [29,74] found that after grinding, the SG of POFA improved due to the reduction in porosity, evidencing that POFA enriched the porosity of concrete up to 2 wt.% [30]. The density of concrete could be reduced because of the absorption of water via permeable POFA particles. The growth of concrete strength is affected via hydrated mortar’s permeability (Figure 10) [109].

This effect relies on the hydrated paste and the w/c ratio. It is revealed that the upsurge in the content of unground POFA lessened the air-dry densities of concrete the content of Ca(OH)_2_ of hydrated paste, including the bubbles between hydration products and aggregates. Therefore, making a more solid concrete because the unground POFA ash could increase impermeability of concrete via pore refinement [32,39]. Meanwhile, the POFA distinguished the size of pores and condensed concrete’s permeability, making a dense concrete [16]. The total porosity of the POFA mortar is also decreased after the CO_2_ exposed to natural air for 28 days [16,32,61,73]. Even though porosity is different in FA/POFA-based geopolymer concretes, pores initiated in both materials can produce a discharge system for moisture. Porous size can be active in the assembly of these escape systems due to the grinding process reduces the permeability by decreasing the particle size [44]. Unreacted particles disappear after contact to 800 °C, demonstrating that concrete paste with a larger content of POFA inclines to captivate high water content as a result of a high permeability [16,39,73,74,81,99]. Moreover, the low volume of porous Ca(OH)_2_ in the presence of POFA is due to low lime content [29,74]. The supplemental C–S–H gel from pozzolanic reaction could also be made at the Ca(OH)_2_ outflow. Therefore, the concrete matrix became denser with a decrease in porosity [11,29], causing a low diffusion of acid solution inside the concrete matrix. The POFA paste that was carbonated could retain less permeability than the free POFA paste from carbonation because of the deduction of CaCO_3_ formed in the attendance of CO_2_ [1,30]. However, the porosity (ε) of the composite membrane was computed through the way reported by previous research [111].

## 6. Fresh Properties

The fresh POFA concrete has a different performance compared to normal fresh concrete. POFA concrete has several properties at the fresh state, such as workability, setting time, segregation, slump loss, bleeding, and shrinkage. All are related to the numerous POFA mixtures properties and generally assessed through J-Ring, T_50cm_ slump flow test methods. The fresh properties of POFA are described in the subsequent subsections.

### 6.1. Workability

Reportedly, the volume of additional 12% water content in POFA and 6% naphthalene-sulfonate-based SP dosage, by weight, is a significant gradient that is necessary for regulating workability and strength [47,75,88]. Various experimental studies reported that POFA has no adverse influence on concrete workability. Nevertheless, workability reduces with the further addition of POFA content [23,27,39], as shown in Table 5. The more replacement of POFA displays a low slump, leading to a low compaction degree that requires more water than normal concrete [20,37]. This is because of the high porousness of POFA particles that retain water and diminishes the free water content required for workability. Segui et al. [112] reported that the high porosity of binder materials with an agglomerated morphology led to reduced workability because of increased water absorbed by large open areas. However, the workability of mortar (flow diameter) decreased from 12.25 to 11.25 as the POFA content increased from 30% to 70% [88]. Furthermore, the increase in fineness of particles adds waster to increase the workability of POFA paste [38,65]. Also, the use of Ca(OH)_2_ content with sand particles of 45 mm size and 0.35 water–binder (w/b ratio) showed good workability [53] studied three sand–cement paste samples to determine differences in. Furthermore, high POFA content increased the viscosity of concrete, reduced L-box, J-ring, slump flow, and augmented T_50cm_ slump flow, segregation index, and V-funnel flow time [51]. The addition of either polycarboxylate ether (PCE)-based superplasticizer (SP) or Glenum 51 at 1.5% by mass of binders enhanced workability due to adequate bonding among aggregate particles [113]. In another study, polymer-based SP’s use at 1.0% by mass of SCMs increased the concrete workability [78]. The un-burnt carbon particles can absorb a substantial SP, detached by reheating POFA at 500 °C for 60 min and reducing workability [52]. However, in terms of physical characteristics, fine micro-sized POFA exhibits low specific gravity (SG), small median particle size, and a large area of the specific surface [52,64]. These properties contribute to the improvement of workability of fresh concrete. No segregation was observed while mixing the concrete and the factor of compaction varied between 0.93 and 0.97. It is reported that the concrete replaced with 0%, 50%, 60%, and 70% POFA rate resulted in modest slump results of 80, 90, 115, and 160 mm, respectively [54,65]. Also, the use of nano-silica in pastes comprising unground POFA condensed the need for water-reducing admixtures and SP to attain concentrate with anticipated workability [91]. The concrete containing 20% POFA was found no opposite effects on the fresh characteristics, including the workability of SCC [51,64]. However, the increase in workability can be decreased due to low carbon and LOI content in a finer POFA. Given that the substitution was by volume, the binder content was reduced because of the lesser SG of POFA than Portland cement.

It is found that POFA concrete is affected by several parameters for instance aggregates quantities, cement hydration, ambient conditions, evaporation, types and interrelation between different materials, moisture contents, mixture proportions and total water content [14,16,19,29,47,57,59,60,75,81,94,115,116]. However, these factors may affect the rate and extent of slump loss (SL) and can be controlled by a ready mixed concrete producer [75]. Reportedly, the inclusion of ceramic powder and POFA binder in OPC concrete with replacement levels of 0%, 10%, 20%, 30%, 40%, 50%, and 60%; with 0.46 w/c ratio and 2% of sodium silicate revealed that the concrete workability without sodium silicate declined the slump value [20,39,53,59,117]. The optimum increment of slump value was recorded at 10% and 40% replacements of POFA. However, at the addition of a 2% superplasticizer to the concrete mix, the slump is obtained between 60 and 180 mm (Table 6) [14,57,78]. Table 7 shows the influence of U-POFA on the decrease of SL. Such an effect may be attributable to the upsurge in the area of the surface of U-POFA particles and the low cement hydration rate and dilution effects.

### 6.2. Setting Time

The POFA concrete setting time usually differs with the degree of fineness of ash and percentage of substitution, informing that the use of-POFA in concrete can perhaps defer the initial and final setting times [47,57,118,119]. At air temperature, 20 °C, ground temperature and climate conditions assume significant roles in the rate at which hydrates [75]. When the cement mixed with water, it made a paste that leads to misplace its softness progressively and lastly goes into a tough-mass. In the setting phase, the cement paste influences the stage of being adequately rigid to resist a certain level of pressure [11]. The period to touch this phase is called a time of setting. Early setting time (<½ h) is defined as the time when the degree of stiffening of a cement mixture is less than that of the time of final setting [16,53,75]. It is also defined as the time passed amid the start of addition, water/cement and when the mix initiates dropping its plasticity [16] (Table 8). The final setting time (<6.25 h) (Table 8). is the time intervened between the start of adding water to the cement and the time at which the mix loses its pliability and achieves an adequate firmness to withstand a certain pressure level [119]. Studies show that POFA mortar has final and early setting times of 10 h and ½ h, correspondingly, at curing temperatures of 20 to 80 °C [52,119]. The inclusion of POFA overdue the paste setting; as such, all setting times augmented with increment in the content of POFA because of the increased volume of water mixed to achieve the anticipated workability (Table 8) [16,39,49,117]. However, other studies showed that the extended times of setting of POFA concrete occurred down to pozzolanic reaction among calcium hydroxide and POFA; such reaction is less than the hydration of cement [29,32].

Furthermore, permeable POFA particles captivated water having no such contribution in hydration reaction, increasing the setting time of pastes [22,78]. Reportedly, the large replacement of POFA contents up to not more than 6.25 h (as prescribed ASTM C 150 [118]) can lead to reducing C_3_S and may not increase the setting time of POFA concrete [11,30]. It was also found that the coarse particles of POFA can delay the setting time on account of the high volume of water engrossed by POFA, leading to delays in the hydration process.

### 6.3. Segregation and Bleeding

Quite a few studies found a little segregation in all concrete mixes contained numerous POFA contents [14,37,39,43,47,75,92,117]. The research reported that concrete mixture with 10% and 15% POFA were highly stable and had a visual-stability-index (VSI) results of “0” and “1”, indicating that a lack of sign of segregation except quite a minor bleeding, respectively [92,94]. It is also revealed that the addition of POFA over prompted segregation and bleeding [94]. Previous researches reported that the inclusion of POFA content not merely enhanced the workability of cement and did not cause segregation but also significantly reduced the bleeding [30,53,117]. Alsubari et al. [53] stated that all mixtures of different POFA concrete fulfilled the necessities of segregation resistance and passing ability as per EFNARC [92]. The high content of modified treated-POFA (MTPOFA) exhibited increased concrete viscosity, leading to a low slump, T50 cm flow time and J-ring tests increased segregation index [51]. In the slump flow test, adding 50% MTPOFA in the SCC mix design did not lead to segregation or bleeding in the concrete; moreover, the J-ring test indicated the lack of blocking and the increased segregation index with increasing MTPOFA replacement level [16,20,52]. Moreover, the slump flow reduced with a larger content of POFA and a low volume of water content [81,94]. Also, the high amount of super-plasticizer might cause bleeding and affect concrete strength [75]. However, no studies have inspected the influence of POFA on bleeding in self-compacted concrete. Particles of POFA are likely more permeable and possess a larger area of specific surface than (Table 9) [88,94].

## 7. Curing Regimes of POFA

To study the effect of curing on solidified concrete characteristics, POFA-based concrete samples are frequently exposed to two different curing conditions, known as air and water curing. The strength of POFA concrete at the two curing conditions is explained in Table 10 and studied in the following sections.

### 7.1. Water Curing

**Figure 11:**
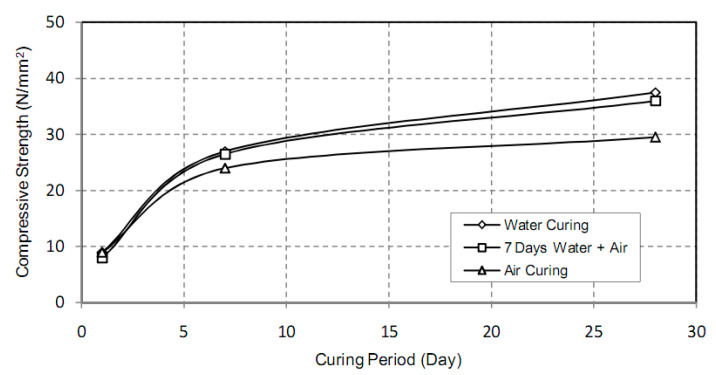
Influence of curing on the POFA concrete strength [57]. Reprinted with permission from Awal et al. [57].

For concrete to realize potential strength and durability, it should have adequate water content for the cement hydration and a temperature that’s tributary for maintaining this chemical action at a fast and continuous rate [47,75]. All test samples should be stowed at 30 °C in the casting room and after 24 h should be de-molded for water-curing [47]. In water curing, sufficient time is allotted. Later, the concrete should be left, and the strength will be increased rapidly at a time up to seven days within which the concrete humid-cured for one week is almost 50% more solid than the un-humid-cured concrete (Table 10 and Figure 11) [57]. In POFA concrete, water curing was greatly influences the strengths, creep, and DS; this curing condition is mainly beneficial in icy weather or when attempting to attain quick strength improvement [15,16,52,120]. Reportedly, water curing improves the properties of pozzolanic materials and enhances their strength by 20% when 20% of the POFA binder is replaced in the mix of concrete [19,25,26,30,44,57,120].

### 7.2. Air Curing

The POFA concrete cured deprived of high heat can be used to other zones outside precast-concrete elements [47,57,121]. Moreover, POFA concrete may accomplish high early compressive strength when cured inside the oven, rather than undergoing natural curing [23,120]. Reportedly, in air curing, the strength of POFA increases up to 98% from seven to 28 days at elevated temperatures [122] (Table 10). For instance, the compressive strength of air-and-water-cured 50% POFA concrete was obtained to be 36.0 MPa for seven days and 41 MPa for 28 days, correspondingly [23,44]. This is anticipated because binder hydration might occur in both the curing of concrete and water-filled capillaries (Figure 11) [57]. Therefore, the development of concrete strength that encompasses pozzolans is extra negatively influenced by trivial curing ages underwater than Portland cement [30,47,60,62].

## 8. Mechanical Properties

After setting, the concrete is required to harden in order to resist live and dead loads sufficiently. Subsequent sections provide a review of the hardened properties of POFA, including flexural, tensile and compressive strengths, the heat of hydration (HoH), modulus of elasticity (MoE), rate of strength development, and stress-strain behavior (Table 10).

### 8.1. Compressive Strength

**Figure 12:**
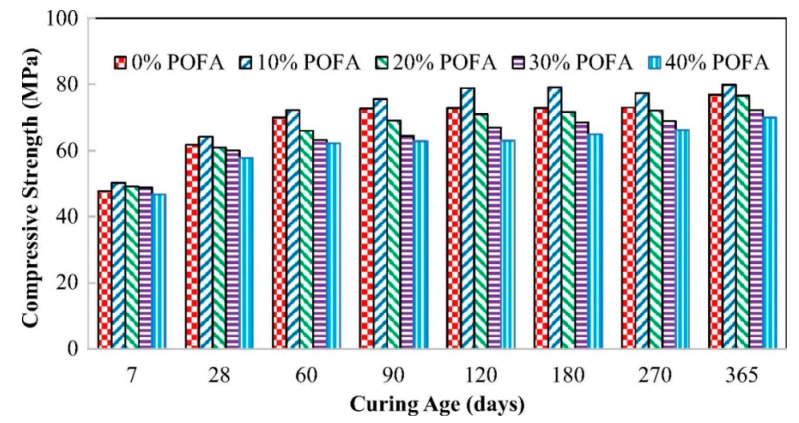
Relationship between compressive strength and POFA with different percentages at different ages [127]. Reprinted with permission from Elsevier [127].

Compressive strength [124] is the strength property of 150 mm-sized cubes examined 28 days [47,75]. Researchers stated that different grades of concrete strength encompassing POFA is effected by the curing time, curing temperature, wet-mixing time, and addition of typical additives [54,65,125]. Many researchers reported that the increase of the content of POFA on the mix of concrete leads to a decrease in the compressive strength [1,20]. It is found that the findings of the 90-day compressive strength of FC enclosing 10% and 20% POFA were 7.17 and 7.06 MPa, respectively [30,66]. Another study showed that the strengths of SCC encompassing up to 70% treated POFA is lower at 3 and 7 days, similar at 28-days, and augmented after three and six months of curing [16,20,51,81]. Also, it is found that the strength at the initial periods of curing significantly decreased in concrete with increasing levels of OPC replaced by ground POFA (MTPOFA) through heat treatment [52,53]. However, the addition of 20–50% unground POFA reported decreasing on the concrete strength and displayed a similar value with 10% unground POFA [39]. These findings could be due to the large and permeable particles of POFA, increases the real w/b proportion in the mix of concrete because of the absorption rate, obtaining in low compressive-strength [22,32]. However, the sample exhibited better or equivalent strength than the reference SCC with increasing curing time. The fine particles of treated T-POFA and MTPOFA promoted the pozzolanic reaction and acted as micro-fillers in the cement paste, leading to enhanced concrete strength [52]. Pozzolanic reaction of TPOFA is considered one of the main parameters that led to the late-age strength. For example, calcium hydroxide (CH) is removed from the prime hydration of cement and interacts with T-POFA. Meanwhile, aluminum and silica join with CH to make C–S–H products [16,51,52,53,94]. This process could enhance the microstructure of SCC and increased its strength. The utmost strength was reported when 10% POFA replaced OPC as a sand replacement; the sample showed 11.31% superior strength than the reference samples. This improvement may be obtained due to the contribution of particles’ fineness and high silica content [126]. Moreover, the slow PA of ground POFA may decrease the early-age strength, but, at the late-age was seen to be more than the identical control samples [32,60]. The 20% ground-POFA content is the most optimum value recommended to attain concrete with greater hardened strength [20]. Sata et al. [23] also observed that concrete with ground-POFA up to 20% had greater strength than normal concrete samples (Figure 12) [127]. This finding is due to the acceptable capability of micro-filling and PA of ground-POFA, mostly contributing to concrete strength improvement at the early stages. Another POFA particles fill the hairy-voids among the cement particles down to their small particles [18]. Other works reported 15% as the ideal ground-POFA content to produce concrete with the extreme increase in strength and it is accredited on the fineness of the particles [128]. Tangchirapat et al. [16] presented that concrete encompassing up to 30% ground-POFA revealed greater 28-day strength than control samples, as shown in Figure 12 and Table 11. It is also found that 40% of ground POFA could be applied in concrete deprived of causing any negative influences on strength [19,20,21]. It is also reported that the reaction between Ca(OH)_2_ and SiO_2_ of ground-POFA and that released from the hydration of cement, in the inclusion of water, via the reaction of pozzolanic and develops a secondary C–S–H, had a positive contribution at motivating the interaction between the aggregates and cement paste, leading to an upsurge of the concrete strength at a long-term [129]. Moreover, the increase of POFA in aerated concrete reduced its compressive strength and exhibited substantial enhancement in strength similar to that 20% replacement level concrete at seven days to 28 days [28]. However, the ability of micro-filling and PA mechanisms of POFA rely on the proportion of w/b in concrete and POFA can be useful when included in concrete with a comparatively small ratio of w/b (Table 11) [29,59].

### 8.2. Splitting Tensile Strength

Reportedly, the concrete comprising up to 30% ground-POFA found that splitting tensile strength (STS) was marginally more significant than that of ordinary concrete. The highest value was obtained in concrete with 20% POFA [11,16,47,51,52,53,75,130,131]. The increase in STS is perhaps because of pore refinement causing by the PA and microfilling ability of ground POFA [27]. Oil palm shell concrete (OPSC) with pozzolanic materials, such as POFA, exhibited 20% lower STS than OPSC without POFA and fly ash [16,61]. OPSC was compared with normal weight concrete (NWC). STS is around 6% of the strength of OPSC and is nearly 8% of the strength of NWC [67]. In another study [120], the STS is 7–8% of the compressive strength of crushed OPSC and 8–14% for NWC [92]. Furthermore, the STS of POFA concrete increased by adding 0–10% POFA and decreasing when the POFA volume exceeded 20% [29,52,53,61,74]. It is also showed that POFA concrete exhibited STS less than that of OPC, and the value increased with increasing level of replacement [26,57,61]. Moreover, the STS of POFA concrete could be overcome by using steel fibers. One alone type of fibers can increase the tensile strength, toughness retention, and impact resistance of concrete because of the binding of the changing zone between the paste and the steel fibers [83,132]. Hence, further investigations must be conducted to study how POFA affects the STS of concrete [15,16,49,57,60,119].

### 8.3. Flexural Strength

Reportedly, the flexural strength of POFA concrete could significantly improve by integrating unlike sorts of small artificial fibers, for instance, polypropylene and PVA, through the linking influence during the macro-and micro-cracking of the POFA concrete texture under flexural load [47,75,92,133]. The use of 20% and 30% POFA-based concrete was exhibited lesser flexural strength than reference samples, but the higher POFA content led to higher flexural strength [26,57,74,90]. It is also found that the concrete encompassing 90% POFA (0.25%, 0.50%, and 0.75% steel fibers with 80 aspect ratio) and OPSC coarse aggregates was increased the flexural strength by 7–18% in comparison with control concrete [83]. However, the specimens with a 65 aspect ratio showed flexural strength greater by 8–12%, respectively, compared with that of control concrete. This finding exhibited that the higher aspect ratio and quantity of steel fibers accomplished great strength. The great surface-area contributes to a robust bond to the binding matrix and excellent resistance to crack spreading. Ranjbar et al. [51,64] saw a decrease in the flexural strength of SCC concrete encompassing POFA, obtaining 6.19 and 6.90 MPa flexural strengths at 7–28 days from curing, respectively. This decrease in the flexural strength can occur attributable to its highly permeable structure that contributes to the condensation of stresses and flagging the interaction among the paste and the aggregate. The increase in POFA content and w/b ratios was displayed to reduce the 1st cracking and flexural strengths [134]. Other investigations reported that the flexural strengths of FC encompassing 20% and 10% POFA at 90 days are 23%and 25% greater than the reference concrete, respectively. This growth may be because of the condensation of the nanostructure caused by the creation of extra C–S–H formed by pozzolanic reaction in POFA [20,23,135].

Furthermore, the use of up to 15% eggshell dust augmented the concrete flexural strength [69]. However, the increase in eggshell content leads to reduce flexural strength. For instance, it is reported that the increase of eggshell content from 0% to 50% at 28 days lessened from 2.86 to 1.0 MPa [14,77]. However, the rupture modulus is computed through the test of flexural strength (a simple beam).

### 8.4. Strength Activity Index

**Figure 13:**
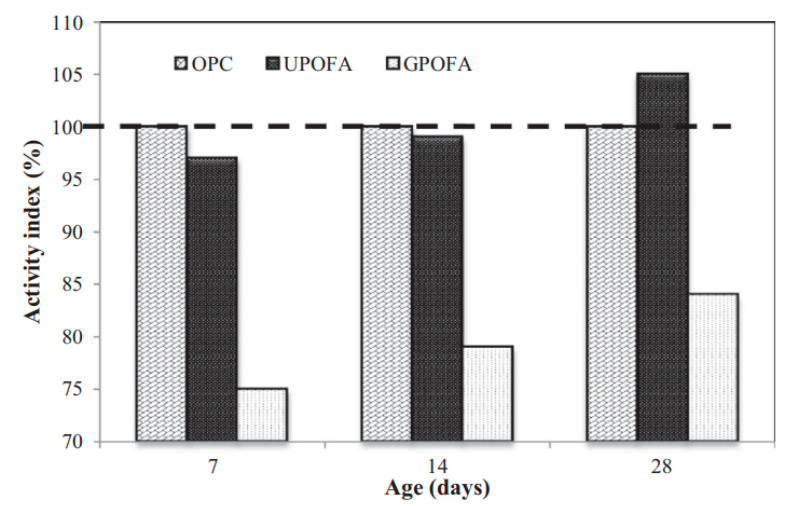
SAI of POFA mortar at different curing period [136]. Reprinted with permission from Elsevier [136].

Strength activity index (SAI, ASTM C 618 [21]) is an indirect technique known as the ratio of the strength of the POFA mixtures to the control strength at every exact remedying time [47,75,92]. The minimum specified SAI value of fly ash is 75% as prescribed by C 618, albeit, there is no guideline established for POFA [16,22,32,49]. The SAI of POFA enriched on account of the increase in the fineness of POFA [60,135]. As shown in Figure 13, the SAI for GPOFA and UPOFA were 75% and 97% of OPC, at seven days, respectively, and UPOFA exhibits 105%, 28 days [136], which led to being bigger than the least necessity rate of 75%, as specified in ASTM C618 [21]. The strength activity catalogs for all mixes are displayed in Figure 13. The level of strength upgrading of OPC depends essentially on its ratio of hydration. The rate also depends on the OPC rehydration and hydration initiated by the POFA pozzolanic-reactivity (PR) in the POFA concrete mixture. Also, Figure 13 portrays that the SAI at 3, 7, 14, 28, and 90 days are 97.3%, 97.6%, 99.3%, 100.7%, and 101.6% [56], led to be larger than the lowest rate of 75% indicated in ASTM C 618 [21]. At 3 and 7 days, the 20% POFA reduced the concrete strength. Thus, this result may be due to the high fineness of POFA as fillers, filling the voids between the sand and the paste, causing more than 97% SAI [16]. At 14 days, the SAI enhanced to more than 99%, attributing to the POFA PR with Ca(OH)_2_. Such a reaction produced C–S–H and improved the strength [23,63]. At an extended curing time of 90 days, the POFA mortar reported a greater SAI rate than 101% [63]. This finding may be because the amorphous aluminous and siliceous minerals energetically reacted with-Ca(OH)_2_, resulting in C–S–H-and hydrated calcium aluminates enhancing the interaction between the paste and the sand [18]. The improvement of these characteristics could lead to increased strength and density of the mixture [18,47].

### 8.5. Pozzolanic Activity

The pozzolanic activity (PA) of POFA is commonly measured with respect to cubes samples’ strength with-and-without pozzolan rendering to ASTM C 311 [137]. The PA relies on the distribution of particle size, silica content, w/b ratio, surface fineness, and possibly improved via the increase in fly ash’s fineness [59]. However, it is reported that the stated lowest PA index of an extremely reactive PCR is commonly 85% [18,31]. In Malaysia, POFA is typically crushed using a crushing apparatus to upsurge its fineness and PA [39,57,74,99]. Despite their advantages, the primary deficiency of POFA in concrete is the postponement in rapid strength growth owing to its small PA that encourages lengthier remedying times [32]. The low initial strength is because of the slow PA of POFA [56]. However, the mixture containing 20% POFA made the utmost strength at 28 days [74]. As the shape of POFA particles increases, it is potential for pozzolanic decreases. Concrete had been cured for one year, had 10% oil palm ash replacement, and sieved via a sieve of 150-μm opening showed 1% lessening in strength than the reference cubes [22,30,49]. The decrease can be accredited to the tiny PA and hydration of POFA, negating the improvement in the strength [54]. Research findings revealed that the more extensive silica content affected the PA through its reactivity with free-lime, thus generating extra C–S–H gels, leading to enhance the concrete strength of POFA [54,65]. The good PA of POFA is obtained when POFA content was replaced up to merely 30% of OPC [16]. Moreover, the increase in LOI content reduces the chemical structure, particularly SOi_2,_ from 69.02% to 59.17%. The improved composition can enrich the PA and significant packing influence, thus improving the strength [16]. Moreover, the decrease of Ca(OH)_2_, MgO, SO_3_, and water absorption in the mixes led to a high PA [29,56,83,113].

### 8.6. Modulus of Elasticity

Modulus of elasticity (MoE) is hugely associated with the concrete compressive-strength such as POFA; a high hydration rate could be detected in a dense POFA texture, leading to a high MoE [35,47,138]. The MoE values are 25–28 GPa at 28 days in ground POFA-based concrete and are 27.5 GPa in concrete with 10–30% OPC replacements [62]. It is likewise revealed that the MoE of concrete encompassing ground-POFA is less and comparable with those of OPC at seven days and 28 days, respectively [25,26,30,32]. In particular, the 20% POFA revealed more excellent MoE than cement at one year’s time. These findings are accredited mainly to enrich the interaction between the paste and the aggregate produced by the PA of POFA [11,51,69]. Though, the influence of POFA concrete on MoE also relies on the aggregate more than paste. The addition of up to 30% ground POFA content exhibited marginally decreasing on the MoE of concrete because of the decrease in coarse aggregate content [49,74]. Moreover, POFA exerted less influence on the MoE of ultra-high performance strength concrete than normal concrete. It is also reported that for ground POFA-based concretes, the MoE values increased with compressive strength by approximately 7% higher than predicted. A similar trend of results was found in fly ash/silica fume-based concrete [139]. The MoE values of OPSC are within 5–11 GPa when the compressive strength is within 24–37 MPa [25,67,83]. The volume of components and stiffness are the main parameters that influence the MoE values of concrete [11,30,31]. This finding might be attributable to the greater modulus of stone aggregates than lightweight aggregates [24,67]. For example, the MoE values of shale aggregates and expanded clay are 5 to 15 GPa. However, the corresponding values for dense natural aggregates, such as quartz, limestone, and basalt, are approximately 60, 80, and 100 GPa, respectively [92]. Another study mentioned that the MoE values of SLWAC vary within 10–24 GPa, which is usually lesser than stone aggregate concrete [41,92]. The study contains 20% POFA shows more excellent MoE value than the control concrete down to the attribution of the use of pozzolanic-ash, leading to being reacted with Ca(OH)_2_ liberated through cement hydration and creates the internal microstructure of dense concrete, thus advancing the MoE of concrete [89]. The hybrid concrete containing 90% POFA with OPSC exhibited lower MoE than that of the reference samples, and this decrease can be overcome by increasing the addition of steel fibers (0.25%, 0.5%, and 0.75%) [83]. Moreover, the MoE of OPS concrete was decreased by integrating oil palm boiler clinker (OPBC) sand. However, the reduction was not substantial when combining OPBC sand up to 37.5%. In addition, 70% OPS’s inclusion with a particle diameter larger than 10 mm formed larger MoE than OPSC with all particles [24,33,67]. Mohammadhosseini et al. [90] reported that the decrease in strength might be accredited to the low MoE value up to 4.9 GPa, using PP fibers, categorizing as soft material. MoE augmented with the addition of POFA caused by the more excellent PA, causing C-H-S gels’ formation [140]. This property does not rely entirely on the alkali-activator dosage but is likewise controlled by the aggregates volume in the mixes of POFA concrete [119].

## 9. Application

**Figure 14:**
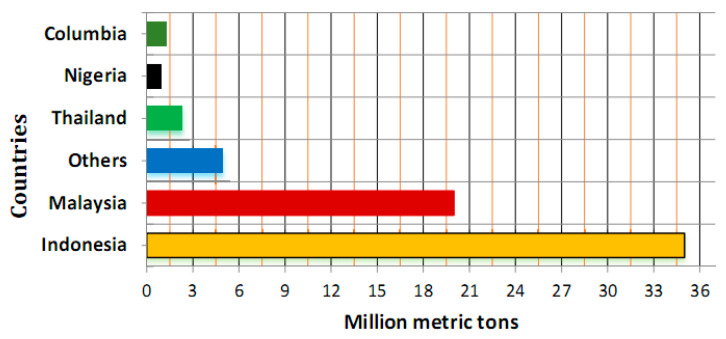
Global palm oil production, 2016/2017 [143]. Reprinted with permission from Karayannis et al. [143].

Normally POFA is used as PCR in the production of concretes and used in several RC applications (Figure 14) with volume, in tons, across the globe, as seen in (Table 12) [11,29,30,32,47,49,51,74,78]. Previous research stated that the expending of POFA in concrete improves the confrontation with sulfate and chloride penetration [22,49,141]. Likewise, the utilization of POFA also improves the additional properties of concrete, for instance compressive and tensile strengths, MoE, and expansion [12,16,19,22,32,49,54,60,62,65]. Meanwhile, water permeability, DS, and w/b are reduced [20,49,62,72,73]. The inclusion of POFA in the formulation of POFA concrete has been so limited, where merely research about POFA cement and concrete has been completed [16,44,73]. The other ash formed from the palm oil mill is boiler ash [12,28]. Boiler ash is merely utilized on roads and mills and ground in plantations [12,16,24,30].

Moreover, POFA ash can potentially fabricate unfired green blocks/bricks that can decrease carbon emission, thus making a maintainable construction element that could avoid and manage pollution and environmental deprivation [11,86]. The constituents of boiler ash comprise silicon, potassium, and phosphorous, are applicable for use as fertilizer and stabilizers in cement and concrete [30,57,82]. The SEM analysis of boiler ash exhibits that the microstructure of boiler ash is similar to POFA. Also, OPS could be utilized as aggregate in the production of lightweight, small footbridges and low-cost house concrete structures that are placed close to the coastal area that has a yearly rain of approximately 2500 mm, air heat of 23–32 °C, and RH of 72–91% [20,39,82]. For clarity, POFA ashes/binders/fillers are commonly dropped in open spaces, resulting in traffic and health hazards and environmental pollution issues [11,52]. Given its wealth and excellent pozzolanic characteristics, dozens of investigators have assessed its potential as a building material [11]. POFA is a promising partial PCR material for future housing construction developments [142]. However, further study on the addition of boiler ash for producing geopolymer material is highly imperative.

## 10. Conclusions

The majority of preceding research concentrated on POFA concrete characteristics, such as their high compressive strength and pozzolanic activity, rather than on the micro-fine morphological properties. Most of these studies also ignored some components. For instance, artificial and natural fibers and alkaline activator solutions affect the strength of the POFA concrete matrix. Further, a binder paste that contains a small size of carbon nanofiber reveals excellent stability and sensitivity properties in POFA concrete. The subsequent noticeable conclusions can be strained based on the sightings from this study review about palm-oil-fuel-ash in a mortar/concrete. The inclusion of POFA as a partial cement replacement in a concrete composite could resolve the dumping and ecological issues produced by the PO industry’s dust, reduce the ecological hazards assumed by the OPC plants, and decrease the CO_2_ emissions in the air and the cement cost. The short-term properties of POFA are auspicious for the making of concrete. Based on this review study, the fineness of POFA is acted as a strong character in concrete. However, the great fineness of POFA increases its micro-filing and PA ability, thereby leading to enhancing the mechanical and durability characteristics of concrete. POFA-based eco-efficient concrete composite presents a similar and, from time to time, a superior recital than normal concrete in withstanding aggressive environments. More investigation is recommended to validate the valuable influences of POFA on numerous concrete properties, and therefore inspire the inclusion of POFA in concrete production.

Further investigations are highly-imperative to prolong the utilization of POFA in SCC concretes. Albeit, POFA has a potential binder as a PCR up to a particular substitution level of OPC deprived of initiating any negative influence on the concrete’s mechanical properties. In the conclusion of this review study and to approve the favorable influences of POFA on some short-term properties of concrete, making POFA as an alternative PCR in concrete, several research investigations are recommended for future studies:

- To investigate the influences of POFA on the slump loss and plastic shrinkage as well as the air content of concrete and examine the influences of POFA on the rheological characteristics, such as plastic viscosity and yield stress of concrete.
- To examine the impress of POFA on the bond, tensile, fatigue, impact, shear, and flexural strengths of concrete; and to study the feasibility of POFA concrete in resisting aggressive environment.
- To study the influences of POFA on the autogenous, creep, water absorption, and shrinkage.
- To investigate the characteristic of high fineness POFA in order to improve the microstructure that results in a highly impermeable matrix, and to prove the potential use of POFA materials in the making of ultra-high-strength and SCC concretes, to expand the strength of POFA in a hardened state using fibers.
- To further survey the possible applications of POFA in building green structures and future maintainable cities with a decreased carbon footprint.

## Figures and Tables

**Table 1:** POFA chemical compositions.

Ref.	Chemical Composition (%)								
	SiO_2_	CaO	MgO	Na_2_O	K_2_O	P_2_O_5_	SO_3_	LOI	SiO_2_ + Al_2_O_3_ + Fe_2_O_3_
[51]	64.17	5.8	4.87	0.18	8.25	-	0.72	-	74.23
[16,52,53]	66.24	5.21	4.83	0.16	6.7	-	0.53	4.41	73.69
[54]	62.6	5.7	3.52	-	9.05	-	1.16	6.25	75.37
[55]	51.18	6.93	4.02	0.06	5.52	4.10	0.36	21.60	59.20
[49]	55.50	12.40	4.60	0.00	0.00	-	2.30	7.90	70.30
-	65.01	8.19	4.58	0.07	6.48	4.69	0.33	2.53	75.10
[16]	59.0	11.00	3.50	-	-	-	-	10.10	67.50
-	63.2	10.10	0.60	-	5.80	-	0.20	13.50	70.70
[56]	66.91	5.56	3.13	0.19	5.20	3.72	0.33	2.30	79.10
[57]	59.62	4.92	4.52	0.76	7.52	3.58	1.28	8.25	67.20
[58]	53.50	8.30	4.10	1.30	6.50	2.40	-	18.00	56.50
[59]	79.30	2.79	1.21	-	3.23	2.32	0.45	-	89.50
[60]	65.30	6.42	3.08	0.36	5.72	-	0.47	10.05	69.80
	43.60	8.40	4.80	0.39	3.50	-	2.80	18.00	59.70
[61]	61.85	5.09	2.79	0.10	5.09	3.32	0.28	9.88	72.90
-	67.09	5.58	3.06	0.11	5.45	3.62	0.32	2.20	79.10
[62]	65.30	6.40	3.00	0.30	5.70	_	0.40	10.00	69.70
[63]	42.50	11.00	7.10	7.00	0.40	5.70	2.20	20.90	45.80
[30]	58.30	6.72	3.69	-	8.40	-	0.96	7.34	74.80
-	49.20	7.50	3.93	0.90	5.30	6.41	1.73	13.85	60.40
[16]	65.30	6.40	3.00	0.30	5.70	-	0.40	10.00	69.70
[64]	63.60	7.60	3.90	0.10	6.90	-	0.20	9.60	66.60
-	53.82	4.24	3.19	0.10	4.47	3.01	2.25	10.49	64.00
[11,15]	64.50	7.80	3.70	0.20	6.60	-	0.10	9.40	67.70
	62.80	7.70	3.60	0.10	6.50	-	0.30	9.70	66.30
	63.60	7.60	3.90	0.10	6.90	-	0.20	9.60	66.50

**Table 2:** Physical properties of OPC, treated POFA, and ground POFA [11,29].

Property	OPC	Ground POFA	Treated POFA
Maximum size (mm)	-	-	-
Median particle size, *d*_50_ (µm)	10–20	7.2–10.1	54.3–183
Water absorption (%)	-	-	-
Color	Grey	Dark grey	Grey
Passed on in a sieve No. 325, with 45 µm, (%)	92	96	100
Surface area, (m^2^/g)	–	104	–
Relative density	3.16	2.04	2.20
Soundness, (mm)	0.45–1	1	0.5–2.6
Strength activity index (%)	-	78.6–115	-

**Table 3:** Physical properties POFA.

Specific Gravity	Blain Fineness (m^2^/kg)	Retained on 45 µm Sieve (%)	Strength Activity Index (%) 7, 28 days	Median Particle Size *d*_50_ µm	Ref.
2.42	493	10.50	-, 112	-	[65]
2.2	-	9.0	-	-	[66,67]
2.56	-	-	-	1.10	
2.42	-	-	98	10.89	[68]
2.56	-	-	-	1.068	
2.15	-	-	-	17.1	[51,64]
1.81	-	-	-	10	
2.59	-	-	-	2.06	[69]
2.53	-	1.70	105, 109	10.70	[49]
2.42	435	-	-	15.76	[55]
2.50	1694	-	-	2.45	
2.50	1438	-	-	2.99	
2.56	1775	-	-	2.06	
2.36	670	-	-	15.60	[40]
2.48	1490	-	-	2.10	
2.42	493	4.98	79, 93	14.58	[57]
2.78	670	10.60	-	-	[59]
2.39	1228	4.30	-	12.30	[60]
2.22	719	13.70	-	13.00	
2.05	660	34.80	-	30.80	
2.22	520	-	-		[70]
2.42	540	-	-	22.52	[61]
2.56	450	-	-	22.53	
2.36	234	-	-	15.60	[40]
2.48	1800	-	-	2.10	
2.50	-	1.20	-	9.20	[63]
2.25	1180	1.00–3.00	-	7.20	[33,71]
1.97	-	41.20	-	62.50	[72]
2.17	-	17.10	90, 90	19.90	
2.33	-	1.50	89, 95	10.10	
1.95	-	70.00	-	55.00	[73]
2.15	750	15.00	-	20.00	
2.25	1180	3.00	-	7.20	
2.43	-	1.00	-	8.00	[20]
1.97	-	41.20	-	62.50	[74]
2.33	1244	1.50	-	10.10	
3.14–3.28	314–358	-	-	10–20	OPC
-	-	34 (max)	75 (min)	-	ASTM C618-12a

**Table 4:** Temperature variations caused by HoH in POFA-based concrete.

Ref.	Percentage of RePlacement of POFA, %	Initial Temperature (°C)	Peak Temperature Rise, (°C)	Maximum Temperature (°C)	Time Since Mixing to Peak Temperature (h)
[2]	0	27.2	57.3	-	18
	50	27.1	46.8		24
	60	27.8	44.2		24
	70	26.9	41.5		32
[110]	0	31.0	36.7	-	20
	30	31.1	35.4		28
[32]	0	30.7	30.3	61.0	12
	10	30.4	30.0	60.4	14
	20	30.4	27.3	57.7	15
	30	30.6	24.3	54.9	16
[40]	0	26.0	47.0	73.0	12
	10	27.0	46.0	73.0	13
	20	28.0	44.0	72.0	14
	30	28.0	40.0	68.0	15

**Table 5:** Influence of POFA on the concrete workability.

Percentage of Replacement of POFA,%	Ground-POFA		Unground-POFA		Ref.
	Slump, mm	Compacting Factor, %	Slump, mm	Compacting Factor, %	
0	200	0.970	150	0.975	[114]
10	200	-	150	0.970	
20	180	0.950	140	0.965	[23]
30	185	0.930	130	0.960	
40	0	-	130	0.955	[39]
50	0	-	120	0.950	

**Table 6:** Mix characteristics of POFA and OPC concrete [54].

Materials	OPC Concrete	Percentage of Replacement of POFA, %		
		60%	50%	70%
POFA (kg/m^3^)	-	256	213	299
OPC (kg/m^3^)	427	171	214	128
Fine aggregate (kg/m^3^)	787	787	787	787
Coarse aggregate (kg/m^3^)	961	961	961	961
Slump (mm)	160	110	140	80
Water (kg/m^3^)	205	205	205	205

**Table 7:** Influence of slump loss with ultra-fine POFA [60].

Slump (mm)				
Time (Min)	HSC	HSC20	HSC40	HSC60
7	190	210	225	230
30	150	190	210	205
60	135	175	195	190
90	125	155	160	180

**Table 8:** POFA concrete times of setting [20,38,39].

Percentage of Replacement of POFA, %	Early Setting Time, Min		Final Setting Time, Min	
	Ground POFA	Unground POFA	Ground POFA	Unground POFA
0	250	125	390	195
10	255	130	395	225
20	275	130	420	240
30	290	140	445	250
40	310	150	460	270
50	-	160	-	280

**Table 9:** Influence of POFA particles on segregation resistance [59].

Mix. No.	W/B	Percentage of Replacement of POFA, %	Ability of Filling			Ability of Passing	Resistance of Segregation	
			T50 cm Spread Time (s)	V-Funnel Flow Time (s)	Slump Flow (mm)	J-Ring Flow (mm)	Segre. Factor (%)	Segre. Index (%)
1	0.5	0	1.10	1.50	655	655	25.2	23.2
2		5	1.13	1.89	655	645	23.8	22.0
3		10	1.43	2.37	650	630	15.7	15.7
4		15	1.81	2.66	630	610	10.9	11.3
5	0.6	0	0.57	1.35	650	635	19.1	21.3
6		5	0.58	1.64	640	635	16.1	20.2
7		10	0.88	1.99	610	600	12.7	14.7
8		15	0.97	2.52	600	585	10.8	10.2

**Table 10:** Properties of oil palm shells as construction materials [123].

Properties	Oil Palm Shells
Specific gravity	1.17–1.37
Bulk density (uncompacted) (kg/m^3^)	510–550
Void ratio (compacted) (%)	57
Void ratio (uncompacted) (%)	63
Absorption of water at 24 h (%)	21–33
Flakiness index (%)	65
Value of aggregate crushing (%)	5–10
Thermal conductivity (W/mc)	0.19
LOI (%)	98–100
Value of aggregate impact (%)	4–8
Thickness of shell (mm)	2–8
Los Angeles abrasion value (%)	3–5

**Table 11:** Compressive strength with different setting times.

Percentage of Replacement of POFA, %	w/b Ratio	SP (%)	Slump (mm)	Compressive Strength (MPa) 28 d	Final Setting Time (Min)	Initial Setting Time (Min)	Ref.
0	0.28	2.3	160	48.0	-	-	[22]
25		2.3		57.5			
0		2.0		46.0			
50	0.48		-	41.0	-	-	[65]
60		2.0	115	36.0			
70		2.0	90	28.0			
0		2.0	80	75.0			
10		-	-	79.3			
20	0.35	-	-	77.3	-	-	[40]
30				72.8			
40				66.5			
0	0.27	2.2	190	91.4	285	140	[55]
20		2.2	210	98.3	385	230	
40		2.2	225	104.2	460	270	
60		2.2	230	98.1	555	350	
0	0.35	0.40 0.75	-	75.0	-	-	[40]
10				80.2			
20				77.3			
30				72.8			
40				66.5			
0				68.9			
10				76.5			
20	0.35	0.80	210	58.3	-	-	[16,74]
30		0.83		48.3			
40		1.0		43.3			
0		7.6		65.0			
10	0.30	7.5	210	67.5	-	-	
20		9.0	220	67.0			
30	0.40	18.5	220	65.5	-	-	[57]
0		1.0	60	-			
50		1.0	45	39.5			
0	0.32	1.16	245	58.5	-	-	[16]
10		1.24	250	59.5			
20		1.56	240	60.9			
30		2.11	250	58.8			
0	0.67	-	-	42.8	180	114	[65]
10				42.0	180	120	
20				40.6	195	124	
30				38.7	210	130	
40				33.8	210	133	
0	0.28	1.41	200	85.0	-	-	[23,96]
10		1.52	200	81.0			
20		2.11	185	86.0			
30		3.02	185	80.0			
0	0.71	-	75	26.1	390	250	[20]
20	0.73		65	23.9			
40	0.74		70	20.7			
55	0.75		90	18.1			
0	0.70	-	65	31.9	395	265	[32]
10			55	31.9	420	275	
20			60	31.6	445	290	
30			60	30.1	460	310	
40			60	27.5			
0	0.60	-	150	35.5	195	125	[39]
10			150	35.5	225	130	
20			140	29.5	240	130	
30			130	25.5	250	140	
40			130	20.2	270	150	
50			120	17.8	280	160	

**Table 12:** Applications of POFA.

Percentage of Replacement of POFA, %	Applications	Ref.
POFA filler POFA ash POFA binder	To upsurge the concrete strength	[16,43,53,78,89]
	To use as an effective polymer concrete filler	[107]
	To reduce the total temperature rise	[66]
	To improve surface resistance and water permeability of concrete	[49]
	To construct roads, ground in the plantations and mills	[12,16,30,82]
	To produce unfired green bricks	[57,139,144]
	In produce foamed concrete	[24,25,66,145]
	To produce aerated concrete	[73,81,139]
	To produce lightweight FC for non-structural building material	[24,25,33,66]
	To construct footbridge and low-cost house	[1,14,70]
0% and 20%	The 20% POFA in concrete attained in the reduction of DS.	[26,90]
0%, 5%, 7.5%, 10%, 12.5%, 15% and 17.5%	The 12.5% of POFA substituted on OPC displays the enhancement in strength.	[8,146]
0%, 10%, 30% and 50% 0% and 70%	The 10% of POFA illustrates the greater strength than reference at the age of 3 months.	[39,64,143]
0%, 50%, 60% and 70%	The strength of concrete encompassing a high POFA content explains the lesser strength. The high content of POFA reduced workability but improved with the use of a super-plasticizer.	[57]
0%, 5%, 10% and 15%	Workability indicates a significant decrease with the addition of a high volume of POFA. The 15% POFA substitution demonstrates a higher strength similar to normal concrete samples.	[30,64,120,126]
0%, 50%, 60% and 70%	The abilities of passing and filling are superior to reference. 70% of OPC substitution was recommended to make durable concrete.	[51,52,53,61]
0%, 10%, 15% and 20%	The increments of the content of POFA contribute to a reduction in initial hardened properties, but the SCC strength comprising POFA was equivalent.	[51,64]
0%, 30% and 60%	The replacement of POFA, shows weak concrete workability with an acceptable limit. Higher content of POFA substitution presents a reduction in strength.	[66,67]
10%, 20%, 30%, 40% and 50%	The 20% inclusion of POFA into FC exhibits the suitable strength of FC for non-structural concrete applications.	[24,25,66]

## Data Availability

Data sharing not applicable

## Abbreviations

AIR	Acoustic insulation resistance
AE	Aggressive environment
ASR	Alkali-silica reaction
CNP	Calcined natural pozzolan
CAPOFA	Calcium salt palm oil fuel ash
DS	Drying shrinkage
EDX	Energy dispersive X-ray
FELCRA	Federal land consolidation and rehabilitation authority
FELDA	Federal land development authority
FA	Fly ash
GFC	Foamed concrete
GGBS	Ground granulated blast-furnace slag
HoH	Heat of hydration
ISAT	Initial surface absorption test
LAAM	Los Angeles abrasion machine
MIP	Mercury intrusion Porosimetry
Mt	Million tons
MTPOFA	Modified treated-POFA
MoE	Modulus of elasticity
NWC	Normal weight concrete
OPC	Ordinary Portland cement
PO	Palm oil
POC	Palm oil clinker
POFA	Palm-oil-fuel-ash
PCR	Partial cement replacement
PSA	Particle size analyzer
PCE	Polycarboxylate ether
PA	Pozzolanic activity
PFA	Pulverized fuel ash
RCPT	Rapid chloride penetration test
RoCA	Rate of capillary absorption
R	Raw
RC	Reinforced concrete
RISDA	Rubber industry smallholders development authority
SEM	Scanning electron microscopy
SL	Slump loss
SG	Specific gravity
STS	Splitting tensile strength
SAI	Strength activity index
SP	Superplasticizer
VSI	Visual stability index
